# Purified Diets Lacking Fermentable Fiber Reduce Microbial Diversity, Alter Epithelial Transcriptome, and Exacerbate Colitis

**DOI:** 10.3390/nu18060891

**Published:** 2026-03-11

**Authors:** Emma Griffith Thomas, Beulah Favour Ortutu, Jacob Connor Watson, Ethan Ong, Kaitlyn Eileen Blankley, Angela Meaurio Martin, Smriti Shankar, Dongmei Zhang, Devon Joseph Boland, Chia-Shan Wu

**Affiliations:** 1 Department of Nutrition, Texas A&M University, College Station, TX 77840, USA; emma_thomas110702@tamu.edu (E.G.T.); beulah@tamu.edu (B.F.O.); watsonj1700@tamu.edu (J.C.W.); theoracletamu@tamu.edu (E.O.); kblankley@tamu.edu (K.E.B.); angela.meaurio@tamu.edu (A.M.M.); 2 Integrated Metabolomics Analysis Core, Texas A&M University, College Station, TX 77843, USA; smriti.shankar@tamu.edu; 3 Texas A&M Institute for Genome Sciences and Society, Texas A&M University, College Station, TX 77843, USA; dongmei@tamu.edu (D.Z.); devonjboland@tamu.edu (D.J.B.)

**Keywords:** dietary fiber, microbiome, colonocyte transcriptome, gut inflammation

## Abstract

**Background/Objectives**: Dietary fibers play key roles in shaping gut microbiome and intestinal homeostasis. While purified diets offer experimental precision and reproducibility in rodent models, they omit the complex mixture of fermentable and non-fermentable fibers found in grain-based chow diets. We hypothesized that excluding fermentable fiber impairs intestinal homeostasis by reducing microbial metabolites and altering the colonic epithelial transcriptome, thereby increasing susceptibility to inflammation. **Methods**: Wildtype male C57BL/6 mice were maintained on either a standard grain-based chow diet or a purified low-fat diet (LFD) containing 5% non-fermentable cellulose for ten weeks. Fecal microbiomes, short-chain fatty acid (SCFA) profiles, and colonic epithelial transcriptomes were analyzed. A separate group was challenged with dextran sodium sulfate (DSS) following a five-week dietary intervention to compare colitis severity between the two diet groups. **Results**: Relative to mice fed the grain-based chow, those consuming the purified LFD (containing only non-fermentable cellulose) showed decreased gut microbial diversity and significantly lower SCFA levels. These changes were accompanied by marked differences in colonic epithelial cell transcriptomes. In LFD-fed mice, the top upregulated gene networks included ribosomal pathways and MHC complex protein binding, suggesting increased growth and gut inflammation. The most downregulated pathways included mineral absorption, actin and tubulin binding, and membrane organelle assembly, indicating major alterations in cellular structure and transport. LFD-fed mice also exhibited increased colonic expression of S100a9, a gut inflammation biomarker, and more severe disease symptoms when challenged with DSS compared to chow-fed mice. **Conclusions**: Fermentable fibers are one of the factors contributing to intestinal homeostasis and mitigating the severity of ulcerative colitis.

## 1. Introduction

It is widely acknowledged that the gut microbiome acts as a vital link between dietary nutrients and the development of chronic diseases [1,2]. Beyond the quantity and duration of exposure, the specific types of food and the degree of processing significantly impact microbial diversity. Different dietary components selectively promote the growth of bacterial groups with specific metabolic functions [2,3,4,5]. Thus, diet shapes the gut microbiota’s composition and function, which in turn impacts the host’s intestinal and metabolic health.

When comparing different diets and their effects in animal models, often a grain-based chow diet is used as the control, especially in high-fat diet-driven metabolic syndrome models. However, variations in the composition of chow diets, such as levels of phytochemicals, micronutrients, and fibers, could impact experimental results [6]. On the other hand, while semi-purified low-fat diets (LFD) that are matched in micronutrients to experimental high-fat diets have been developed as appropriate control diets in diet-induced obesity studies [7], the nature of fiber also influences gut microbiome and host physiology [8,9].

Based on their susceptibility to microbial breakdown, dietary fibers are generally categorized as either fermentable or non-fermentable. Highly fermentable fibers, such as inulin, pectins, fructans, gums, and resistant starch, serve as substrates for gut microbes. This fermentation process produces short-chain fatty acids (SCFAs), which are essential for maintaining intestinal barrier function, regulating the immune system, and supporting metabolic health [8,10,11]. In contrast, non-fermentable fibers (including lignin, cellulose, and hemicellulose) remain largely intact as they pass through the colon, resisting bacterial degradation. Interestingly, recent studies have reported reduced microbial diversity in mice fed with purified diets compared to grain-based chow diets [12,13,14]. In addition to fiber content, chow diet also contains numerous bioactive compounds naturally found in plants that are absent from purified diets, such as phytochemicals (plant sterols, polyphenols), and phytoestrogens in soybean-based chow (isoflavones from soy) [6,7]. These “non-nutrient” bioactive compounds also affect microbiota, intestinal barrier function, and inflammatory pathways. The direct impact of purified diets on the intestinal epithelial cells lining the colon, which serve as the interface between the diet and the gut microbiota, remains unclear.

To address this knowledge gap, we compared wildtype C57BL/6 male mice fed a standard grain-based chow diet, which contains mixed dietary fibers from wheat middlings, ground wheat, dehulled soybean meal, and ground corn, to mice fed a purified LFD containing 5% non-fermentable cellulose as the only fiber source. We hypothesized that excluding fermentable fiber impairs intestinal homeostasis by reducing microbial metabolites and altering the colonic epithelial transcriptome, thereby increasing susceptibility to colitis. We compared gut microbiome composition, fecal SCFA levels, and the colonic epithelial cell transcriptome between the two diet groups. To assess the consequences of an altered gut microbiome and transcriptome, we evaluated susceptibility to experimental ulcerative colitis in both groups.

## 2. Materials and Methods

### 2.1. Animal

Male C57BL/6J mice, aged approximately 5 weeks, were obtained from The Jackson Laboratory (Bar Harbor, ME, USA) and acclimatized at the Texas A&M University animal facility. The animals were maintained under controlled environmental conditions (22 ± 2 °C; 12 h light/dark cycle) with unrestricted access to water and a standard chow diet (Teklad 2018, Inotiv, Lafayette, IN, USA). The chow diet contains 6% fat from soybean oil and mixed dietary fibers from wheat middlings, ground wheat, dehulled soybean meal, and ground corn. At 12 weeks of age, mice were randomly assigned to two groups: one group received a semi-purified, compositionally defined low-fat diet (LFD, D12450J, Research Diets Inc., New Brunswick, NJ, USA), and the other remained on a chow diet. The refined diet D12450J contains 4.3% fat (2.3% from soybean oil and 2% from lard) and 5% cellulose as a fiber source.

Diet composition is summarized in Table 1. All experiments followed the ethical guidelines of Texas A&M University (AUP 2022-0184, AUP 2025-0115).

### 2.2. Experimental Design

Two cohorts of mice were used. In cohort 1 (n = 4 and 6 for chow and LFD groups, respectively; 2 mice per cage), after ten weeks of diet switch, fresh feces were collected in the mornings between 9 and 10 A.M., with 2 pellets stored in DNA/RNA shield buffer (Zymo Research, Irvine, CA, USA) for microbiome analysis, and 5–6 pellets frozen for SCFA analysis. At termination, mice were euthanized, and colons were dissected, flushed with ice-cold phosphate-buffered saline (PBS), and opened longitudinally. The colon strip was cut lengthwise; 1/3 was flash-frozen for protein analysis, and 2/3 was used for the isolation of colonic epithelial cells. Briefly, the strip was incubated with 25 mM EDTA in PBS for 30 min at 37 °C, then transferred to fresh pre-chilled PBS and vortexed. Then, the colon strip was removed, and the cell suspensions were centrifuged for 7 min at 300× *g* at 4 °C to collect the cell fraction enriched in intestinal epithelial cells. The cell pellets were stored at −80 °C until analysis.

In cohort 2 (n = 4 and 4 for chow and LFD groups, respectively; 4 mice per cage), after five weeks of diet switch, mice received 2% dextran sulfate sodium (DSS, 36–50 kDa; MP Biomedicals, Irvine, CA, USA) in drinking water at libitum for 6 days to induce colitis, as we described [15], adapted from Chassaing et al. 2014 [16]. Disease symptoms, including body weight, fecal consistency, and macroscopic fecal blood, were monitored during disease progression (see the figure in Section 3.4 for scoring schemes). Mice were euthanized on Day 7.

### 2.3. Microbiome Analysis

Fecal pellets were sent to Zymo Research for 16S rRNA (V3–V4 region)-based microbiome analysis, following the company’s standard workflow and bioinformatic analysis. The resulting Amplicon Sequence Variants (ASVs) were subjected to further analyses using MicrobiomeAnalyst 2.0 [17].

### 2.4. Fecal SCFA Measurement

Fecal SCFA concentrations were measured with a gas chromatography triple quadrupole mass spectrometry (TSQ EVO 8000, Thermo Scientific, Waltham, MA, USA) as we described before [18]. Briefly, weighed lyophilized feces were extracted in ethyl acetate; final μM concentrations of the quantified metabolites were normalized to mg dried fecal weight.

### 2.5. RNA Isolation and RNA-Sequencing Library Preparation

Total RNA was extracted from frozen colonic epithelial cell pellets using the Zymo Quick-RNA Miniprep kit (Zymo Research) following the manufacturer’s instructions. Total RNA quality was assessed using the Agilent TapeStation (Santa Clara, CA, USA). Samples with RNA integrity numbers (RIN) greater than 8 were used for RNA-sequencing (RNA-seq) library preparation and sequencing, carried out by the Texas A&M Institute for Genome Sciences and Society (TIGSS) Molecular Genomics Core. Briefly, poly (A)-based RNA-seq libraries were prepared using the Illumina Stranded mRNA Prep Kit (San Dieta, CA, USA) and sequenced at 2 × 150 paired-end reads with the S4-XP workflow on an Illumina NextSeq 6000 platform.

### 2.6. RNA-Seq Data Processing and Differential Expression Analysis

Reads were trimmed to remove low-quality bases (Phred score < 20), adapter sequences, and barcode sequences using Trim Galore [19]. Sequences were aligned to the *Mus musculus* transcriptome (Ensembl GRCm39.112) and transcript-level abundances were quantified using Salmon v1.10.2 [20].

Transcript-level estimates were imported into R (v4.5.2) and summarized to the gene level using the tximport package (v1.36.1; Bioconductor v3.21), based on a transcript-to-gene mapping generated from the *Mus musculus* Ensembl GRCm39.112 GFF3 annotation file using GenomicFeatures (makeTxDbFromGFF). Differential gene expression analysis was performed using DESeq2 (v1.48.2) [21]. Genes with low expression were filtered prior to model fitting; specifically, genes were retained for analysis only if they had a raw count of at least 10 in all samples. Statistical significance was assessed using the DESeq2 negative binomial generalized linear model with Benjamini–Hochberg–adjusted *p*-values. Differentially expressed genes were defined as those with an adjusted *p*-value < 0.05 and an absolute log2 fold-change ≥ 0.5.

Functional enrichment analysis was conducted using clusterProfiler (v4.16.0) [22]. Enrichment of Kyoto Encyclopedia of Genes and Genomes (KEGG) pathways [23] and Gene Ontology (GO) terms [24] was performed using the set of significantly differentially expressed genes and a background universe comprising all tested genes. Enriched pathways and terms were considered significant at a BH-adjusted *p*-value < 0.05.

### 2.7. Colon Tissue Extracts and Capillary Wes Analyses

Colon tissues (~30 mg) were homogenized in radioimmunoprecipitation assay (RIPA) buffer (MilliporeSigma, Burlington, MA, USA) containing protease inhibitor cocktail tablet (Thermo Fisher Scientific, Waltham, MA, USA) using a VWR tissue homogenizer (Radnor, PA, USA). The homogenate was centrifuged at 10,000× *g* for 10 min at 4 °C, and the supernatant was transferred to a fresh tube. Total protein concentrations were measured using the Pierce BCA Protein Quantitation kit (Thermo Fisher Scientific).

Capillary Western analyses were performed using a Wes system (Protein Simple, San Jose, CA, USA) according to the manufacturer’s instructions. Protein samples were diluted with 0.1× Sample Buffer (Protein Simple). The final protein concentration used in our assay was 1 mg/mL. Separations were performed using the 12–230 kDa capillary cartridges (Protein Simple). Primary antibodies were first validated and then used at the following dilutions: 1:50 for anti-S100A9 rabbit antibody (73425S) and 1:200 for anti-GAPDH rabbit antibody (2118S). Both antibodies were purchased from Cell Signaling (Danvers, MA, USA). The anti-rabbit detection module, DM-001, including secondary antibody and chemiluminescent reagents, was purchased from Protein Simple. Data was analyzed using the Compass software (Protein Simple); quantifications were obtained using the area under the peak of the protein of interest, normalized to GAPDH, and data were presented as % chow.

### 2.8. Statistical Analysis

Data processing and statistical evaluations were conducted using GraphPad Prism 10.4.2 (GraphPad Software, San Diego, CA, USA). Significance was determined at a *p*-value of less than 0.05. Data are visualized as mean ± standard error (SE) for bar graphs, whereas box plots show the median and 25th/75th percentiles, with whiskers representing the extreme values (min to max).

## 3. Results

### 3.1. Lack of Natural Fibers in Purified LFD Decreased Gut Microbial Diversity

**Figure 1:**
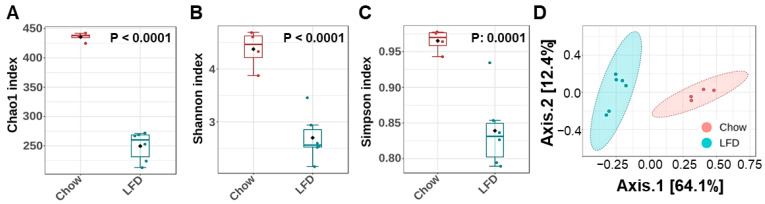
Purified low-fat diet (LFD) decreased fecal microbial diversity. Fecal samples were collected from mice in chow and LFD groups (n = 4 and 6, respectively), after ten weeks of diet switch, and processed for 16s rRNA microbiome analysis. (**A**–**C**) Boxplots showing alpha diversity of the gut microbiome, assessed using (**A**) Chao1 Index, (**B**) Shannon Index, and (**C**) Simpson Index. The significant differences between the diet groups were determined using Student’s *t*-test. Boxplots display median and quartiles of the data points. (**D**) Principal coordinate analysis (PCoA) plot of microbial beta-diversity based on Bray–Curtis’s distance. The fractions of total variance explained by the axes (principal coordinates) were reported as percentages in square brackets.

Since nutritional changes play a large role in shaping gut microbiota composition, we first compared the fecal microbiome of chow-fed mice with that of mice fed a purified LFD using 16S rRNA sequencing. Analysis of alpha diversity indices showed a marked reduction in microbial richness and evenness in the LFD group, as indicated by significantly reduced Chao1, Shannon, and Simpson indices (Figure 1A–C, respectively). Moreover, permutational ANOVA analyses using Bray–Curtis dissimilarities of normalized abundance data revealed clear clustering by diet, with Axis-1 explaining 64.1% of the total variance (PERMANOVA, *p* = 0.008) (Figure 1D).

**Figure 2:**
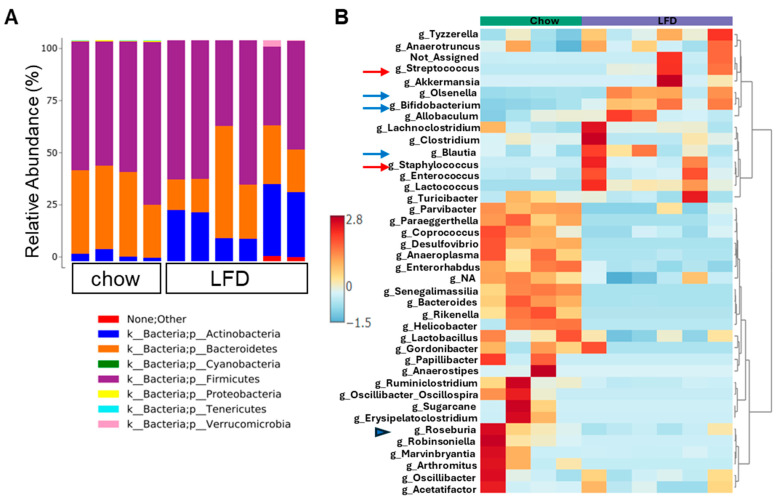
Purified low-fat diet (LFD) altered microbial composition. (**A**) Bar graph showing the relative abundance of the phyla in fecal samples from each mouse. (**B**) Heatmap of microbial community composition at the genus level. Rows and columns represent individual genera and samples, respectively. Color intensity reflects normalized relative abundance.

Taxonomic profiling at the phylum level showed that the gut microbiota in both diet groups was dominated by *Bacteroidetes* and *Firmicutes,* while the *Actinobacteria phylum* was more abundant in the LFD group (Figure 2A). Linear Discriminant Analysis for Effect Size (LEfSe) analysis showed that, at the family level, several differences between the chow and LFD were detected, with *Lachnospiraceae*, *Lactobacillaceae, Ruminococcaceae* and *Provotellaceae* enriched in chow-fed mice (Supplemental Table S1. This excel file contains raw data of effect size and *p*-values from statistical analysis. The group in which the taxa was more abundant is in column B). Moreover, differential abundance analyses of taxa at genus level showed that *Roseburia*, a gut bacterium that ferments soluble fiber into butyrate, were more abundant in the chow-fed mice (Figure 2B, blue arrowhead), consistent with chow diet containing fermentable fiber as substrate. LFD feeding increased the abundance of several genera associated with dysbiosis, including *Streptococcus* and *Staphylococcus* (Figure 2B, red arrows). Interestingly, despite the low fiber content in the LFD, the SCFA-producing taxa such as *Bifidobacterium*, *Olsenella*, and *Blautia* were increased in LFD-fed mice compared to chow-fed mice (Figure 2B, blue arrows), suggesting a switch in bacterial metabolism.

### 3.2. Fecal Levels of Short Chain Fatty Acids Are Decreased in Purified Diets

**Figure 3:**
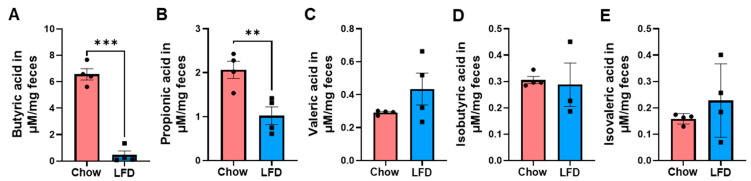
Differences in fecal SCFA concentrations in the chow and LFD groups. Fecal samples collected from chow- and LFD-fed mice (n = 4 for each group) were processed for SCFA measurements. (**A**) butyric acid, (**B**) propionic acid, (**C**) valeric acid, (**D**) isobutyric acid, and (**E**) isovaleric acid. Results were presented as mean ± SE. The significant differences between the chow and LFD groups were determined using Student’s *t*-test. ** *p* < 0.01, *** *p* < 0.001. LFD: Low-Fat Diet.

Next, we assessed SCFA and BCFA levels in feces from mice in the two diet groups. LFD-fed mice exhibited significantly lower concentrations of butyrate (Figure 3A) and propionate (Figure 3B) compared with chow-fed mice, consistent with the lack of soluble fibers in purified LFD. No significant differences were observed in valeric acid concentrations between LFD and chow diets (Figure 3C). In addition, concentrations of the BCFA isobutyric acid (Figure 3D) and isovaleric acid (Figure 3E) were not significantly different between the two diet groups.

### 3.3. Transcripts Altered by Purified Diet in Colonic Epithelial Cells

**Figure 4:**
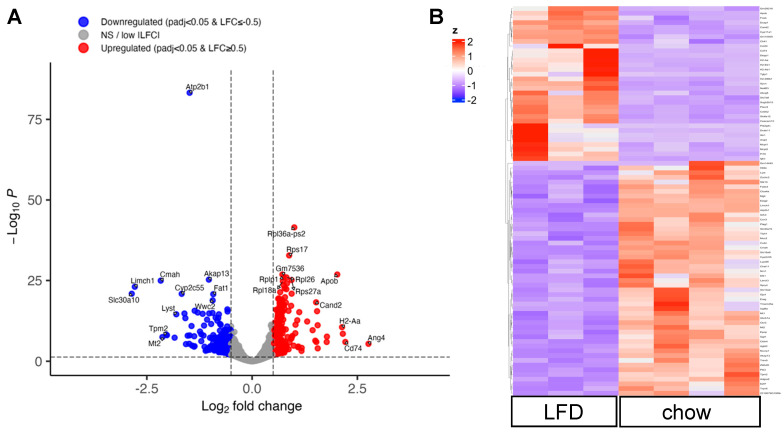
Purified diet modified the transcriptomic landscape in colon epithelial cells. Colon epithelial cells were collected from mice in the chow and LFD groups (n = 4 and 4, respectively) and processed for RNA extraction. One sample from the LFD group failed the quality check (RIN < 8.0) and was not advanced to library preparation and sequencing. (**A**) Volcano plot showing DEGs (up- and downregulated) in colon epithelial cells from the LFD group compared to the chow group. The red dots represent the upregulated genes, and the blue dots represent the downregulated genes. (**B**) Heatmap showing gene expression patterns.

The microbiome and microbial-derived SCFAs exert a strong influence on colonic epithelial cells [25]. To explore the effects of lack of natural fibers in purified diets at the molecular level, we performed RNA-seq analysis on mouse colonic epithelial cells. Volcano plot analysis identified 557 differentially expressed genes (DEGs) between colonic epithelial cells isolated from the chow and LFD groups, based on the criteria of *p*_adjust_ < 0.05 and |log_2_FC| > 0.5 (Figure 4A). Furthermore, a heap map visualization of the DEGs showed distinct patterns of change, suggesting that diet and the gut microbiome exert strong influences on the host cell transcriptome (Figure 4B; list of DEGs in Supplemental Table S2).

**Figure 5:**
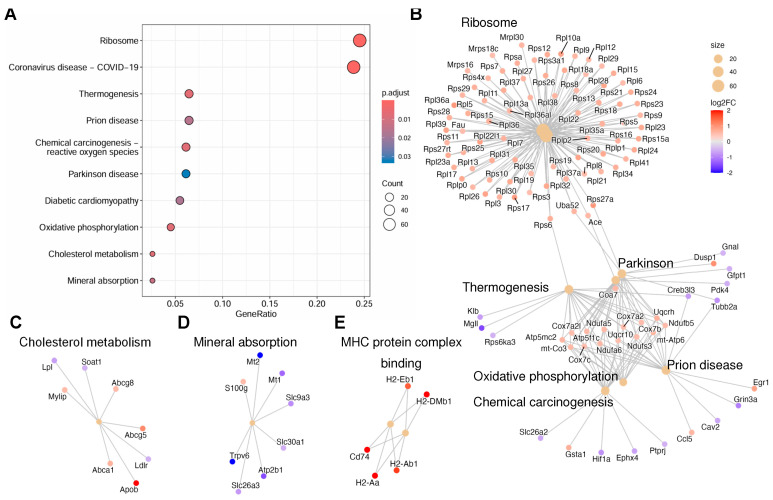
Pathways impacted by purified diets. (**A**–**D**) KEGG analysis of pathways associated with differentially expressed genes in the LFD vs. chow groups (n = 3 and 4, respectively). (**B**–**D**) KEGG network plot highlighting the upregulated and downregulated gene sets. (**E**) Gene Ontology analysis of molecular function, showing upregulated genes in the MHC protein complex binding.

The top four most upregulated genes in the LFD group include *Ang4*, *H2-Aa*, *Cd74*, and *Apob* (Figure 4A, red dots). Angiogenin 4 (*Ang4*) is a member of the ribonuclease A superfamily and plays a significant role in colon epithelial cells, including maintenance of intestinal stem cells, induction of apoptosis, and generation of antimicrobial peptides in response to microbial challenges [26,27]. The *H2-Aa* gene encodes the histocompatibility 2, class II antigen A (alpha), which plays a pivotal role in MHC class II-mediated antigen presentation and the development of T cells. Specifically, its expression in intestinal epithelial cells helps regulate local CD4+ T-cell activity during gut inflammation [28]. This pathway is assisted by CD74, an integral membrane protein that serves as a molecular chaperone for the expression of MHC class II proteins [29]. The *Apob* gene encodes the apolipoprotein B, a crucial component of lipoproteins such as LDL and VLDL that transport cholesterol. In addition, a number of ribosomal genes are upregulated in the LFD group; consistently, the top KEGG pathway impacted by LFD feeding is the ribosome pathway (Figure 5A).

Out of the top eight downregulated genes in the LFD group (*Slc30a10*, *Limch1*, *Mt 2*, *Cmah*, *Tpm2*, *Atp2b1*, *Lyst*, *Cyp2c55*; Figure 4A, blue dots), three are associated with mineral transport and homeostasis. The SLC30A10 is a member of the cation diffusion facilitator superfamily of metal transporters; it is a manganese efflux transporter found on the cell membranes of intestinal epithelial cells and moves manganese out of these cells into the intestinal lumen [30]. The *Atp2b1* gene encodes the plasma membrane calcium-transporting ATPase (PMCA1), which regulates intracellular calcium levels by pumping calcium out of cells. The *Mt2* gene encodes the metallothionein-2 protein that binds to heavy metals such as zinc and cadmium.

Two of the top downregulated genes are involved in actin filament binding: the *LIMCH1* gene codes for the LIM and Calponin Homology Domains 1 protein that is associated with actin stress fibers, while the *Tpm2* gene codes for the beta-tropomyosin. Of note, the *Lyst* gene encodes the lysosomal trafficking regulator (LYST) protein, which is involved in lysosomal transport and maturation, while the *Cyp2c55* gene encodes cytochrome P450, family 2, subfamily C, polypeptide 55. Interestingly, recent studies suggest a correlation between intestinal Cyp2c55 expression and intestinal inflammation [31], while LYST has been linked to cancer and wound healing [32]. Together, the heatmap and volcano plot showed a clear effect of the purified diet on gene expression in colonic epithelial cells.

Next, the identified DEGs were subjected to KEGG pathway analysis. The top pathways significantly impacted by LFD compared to the chow diet include ribosomes, coronavirus disease, thermogenesis, prion disease, chemical carcinogenesis-reactive oxygen species, Parkinson’s disease, diabetic cardiomyopathy, oxidative phosphorylation, cholesterol metabolism, and mineral absorption (Figure 5A). Further analysis by the KEGG network plot showed a remarkable upregulation of ribosomal genes and upregulation of genes in the oxidative phosphorylation pathways (Figure 5B). The *Abcg5* and *Abcg8* genes in the cholesterol metabolism network are upregulated in the LFD group (Figure 5C). These genes encode sterolin-1 and sterolin-2, respectively, and are involved in the excretion of sterols from intestinal cells. Together with the upregulation of *Apob* and downregulation of *Ldlr* (low-density lipoprotein receptor) and *Lpl* (lipoprotein lipase), these data suggest dysregulated cholesterol metabolism in the LFD group. On the other hand, downregulation of genes in the mineral absorption pathway was observed in the LFD group (Figure 5D).

In addition, GO analysis was performed for further functional annotation. The findings from GO analysis mirrored those from the KEGG pathway analysis (Supplemental Figures S1–S3). In the GO analysis of biological processes, the upregulated genes in the LFD group were related to growth and development, including translation and ribosomal biogenesis (Supplemental Figure S1). Of note, in the GO analysis of molecular functions (Supplemental Figure S2), genes involved in MHC protein complex binding were upregulated in the LFD group (Figure 5E). The downregulated pathways in the LED group include actin and tubulin binding, membrane organelle assembly, and apical plasma membrane, suggesting major changes in cellular structure and transport (Supplemental Figure S3, cellular components).

**Figure 6:**
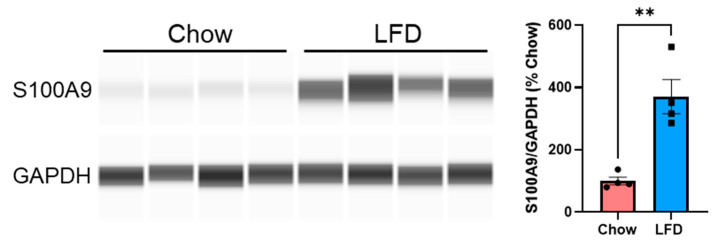
LFD-fed mice exhibited colon inflammation. Colon homogenates from mice in chow and LFD groups (n = 4 in each group) were assayed for S100A9 protein levels using the Capillary Wes System. Data was analyzed with Student’s *t*-test, ** *p* < 0.01.

Upregulation of MHC proteins in colonic epithelial cells has been associated with inflammation and gut dysbiosis [28,33,34]. Thus, we assessed colon protein levels of S100A9, a biomarker of gut inflammation [35]. Indeed, S100A9 proteins were significantly increased in colons of LFD-fed mice compared to chow-fed mice (Figure 6).

### 3.4. Mice on Purified Diet Showed Increased Susceptibility to DSS-Induced Ulcerative Colitis

**Figure 7:**
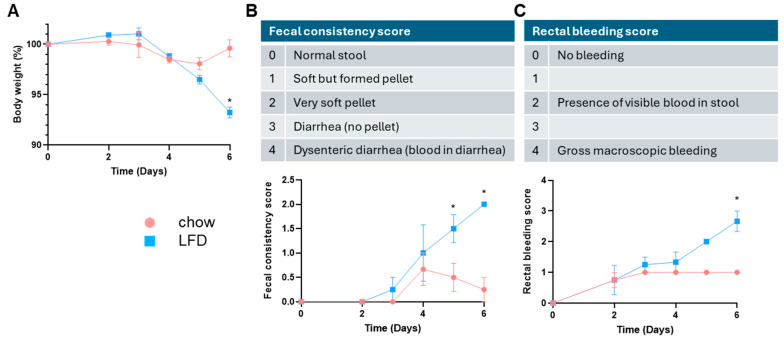
LFD-fed mice were more susceptible to DSS-induced ulcerative colitis. In the second cohort, chow- and LFD-fed male C57BL/6J mice were subjected to 2% DSS in drinking water for 6 days. Disease activities were monitored, including body weight (**A**), fecal consistency (**B**) and rectal bleeding (**C**). Scoring schemes were shown. Data were analyzed with Student’s *t*-test, chow versus LFD groups (n = 4 in each group), * *p* < 0.05.

To explore the functional impact of dysregulated gut microbiome and epithelial cell transcriptomes, we subjected a second cohort of male chow- and LFD-fed mice to an experimental model of ulcerative colitis. Male C57BL/6J mice were treated with 2% DSS in drinking water for 6 days, and disease activity was monitored throughout disease progression. As shown in Figure 7, LFD-fed mice showed exacerbated disease symptoms compared to chow-fed mice, including increased weight loss (Figure 7A), more softening of stool (Figure 7B; a score of 2 indicates very soft pellet with almost no form, while a score of 1 indicates soft but formed pellet), and presence of more visible blood in stool (Figure 7C).

## 4. Discussion

Accumulating evidence indicates that dietary fibers play key roles in shaping the gut microbiome and intestinal homeostasis [8,9]. Grain-based chows contain a wider variety of fibers that provide substrates for microbial fermentation, while purified LFD has only the non-fermentable cellulose as the source of fiber. In line with previous reports [12,13,14], our microbiome data showed that feeding mice a purified LFD significantly reduced microbial richness, evenness, and diversity. Consistent with the limited substrate in purified LFD, the microbially derived metabolites butyric acid and propionic acid were significantly reduced in feces from LFD-fed mice compared to chow-fed mice. The butyrate-producer, *Roseburia*, was also significantly reduced in LFD-fed mice. On the other hand, *Streptococcus* and *Staphylococcus* increased in abundance in the LFD-fed mice; these are opportunistic taxa found in other Westernized diet studies [9,36].

In this study, we observed paradoxical increases in the genera *Bifidobacterium*, *Olsenella*, and *Blautia* in the LFD group; these microbes have been associated with SCFA production [37,38,39]. Despite the increase in these microbes, fecal SCFA levels were significantly reduced in LFD-fed mice, suggesting potential cross-feeding among commensals and altered microbial metabolism. The latter notion is supported by the trend toward increased valeric and isovaleric acids in the feces of LFD-fed mice, suggesting the presence of alternative fermentation pathways. Isovaleric and valeric acids are generated by the fermentation of branched amino acids by the gut microbiota; therefore, under limited fermentable fiber conditions, proteolytic fermentation may be favored over carbohydrate-driven fermentation [40]. Interestingly, a recent study reported increased *Blautia*, *Lachnoclostridium*, *Muribaculaceae*, and *Ruminococcaceae* in reduced-fiber-fed mice compared with chow-fed mice; these were accompanied by increased activities of enzymes targeting mucin glycan linkages [41]. While we did not measure glycan-degrading activities in the current study, it is possible that the changes we observed in microbiota composition could favor mucolytic activity and, consequently, weaken the intestinal barrier. Future studies are required to confirm changes in microbial metabolism and mucolytic activity under low-fiber conditions.

The epithelial cells lining the intestine serve as the interface for interactions among dietary components, microbes, and host tissues [42]. The mammalian colonic lining represents one of the most regenerative surfaces in the human body, undergoing a complete replacement of its cellular architecture roughly every three to five days. This rapid renewal requires a tightly regulated equilibrium: maintaining a robust pool of progenitor cells to drive turnover while simultaneously ensuring a population of specialized, differentiated cells is available for nutrient absorption and barrier maintenance [43]. Through unbiased transcriptomic analysis of colonic epithelial cells, the top pathway upregulated by LFD feeding was ribosomal pathways, suggesting increased epithelial cell growth. Interestingly, in a randomized controlled trial, dietary fiber supplementation with wheat bran (24 g/day) decreased colonic epithelial cell proliferation [44]. Whether LFD feeding leads to increased epithelial cell turnover needs to be further characterized. Notably, we identified upregulation of genes involved in the MHC protein complex binding in the LFD-fed mice. MHC protein complexes on intestinal epithelial cells have been associated with inflammation and gut dysbiosis, triggering CD4+/CD8+ T cell activation [28,33,34]; further characterization of the immune cells in the tissue microenvironment is required to determine the functional implications of the upregulated gene expressions. How fibers modify the expression of MHC proteins and antigen presentation through colonic epithelial cells to affect immunity may present a new angle for future research.

Given that SCFAs regulate epithelial barrier function and mucosal immunity [45], the low butyric and propionic acid levels in LFD-fed mice may predispose them to proinflammatory conditions. Indeed, LFD-fed mice showed increased S100A9 expression in the colon compared with chow-fed mice, and exhibited exacerbated disease symptoms in DSS-induced ulcerative colitis. Together, these data demonstrate the association between cellulose-only purified diets, reduced SCFAs, epithelial transcriptome remodeling, inflammation, and susceptibility to DSS-induced colitis.

Limitations. While fermentable fiber is an important factor differentiating the grain-based chow diet and the cellulose-only LFD in this study, we cannot rule out other factors in the chow diet, such as phytochemicals and phytoestrogens, that could also contribute to the observed effects, particularly in mitigating DSS severity. The 5% cellulose-based LFD was chosen in the study, which differs in bulk from the estimated 15% fiber content of the chow diet and could be a confounding factor affecting gut motility. Furthermore, to elucidate the mechanistic role of fiber in these effects, rescue experiments using fiber-supplemented diets with defined compositions that account for relevant dietary variables are required in future studies.

## 5. Conclusions

In conclusion, our studies highlighted the importance of selecting appropriate control diets in animal studies and that fermentable fibers are key determinants of host physiology and response to ulcerative colitis.

## Figures and Tables

**Table 1:** Composition of diets used in this study. * Crude fiber and neutral detergent fiber are found in the chow diet. Neutral detergent fiber includes cellulose, hemicellulose, and lignin.

Diet	Chow (Teklad 2018)	LFD (D12450J)
Protein source	Crude protein (plat sources)	Casein
Fiber source (gm%)	15 (Crude fiber *)	5 (Cellulose)
Protein (gm%)	18.4	19.2
Carbohydrates (gm%)	44.2 + 15 from fiber	67.3
Fat (gm%)	6	4.3
Energy density (kcal/g)	3.1	3.8

## Data Availability

The original contributions presented in this study are included in the article and Supplementary Materials. Further inquiries can be directed to the corresponding author.

## Supplementary Materials

The following supporting information can be downloaded at: https://www.mdpi.com/article/10.3390/nu18060891/s1. Figure S1: GO-Biological Processes; Figure S2: GO-Molecular Functions; Figure S3: GO-Cellular Components; Table S1: LEfSe results; Table S2: DEG results.

## Abbreviations

The following abbreviations are used in this manuscript:

*Ang4*	Angiogenin 4
*Apob*	Apolipoprotein B
ASVs	Amplicon sequence variants
BCFA	Branched-chain fatty acid
DEGs	Differentially expressed genes
DSS	Dextran sodium sulfate
FC	Fold-change
GAPDH	Glyceraldehyde-3-phosphate dehydrogenase
GO	Gene Ontology
IBD	Inflammatory bowel disease
KEGG	Kyoto Encyclopedia of Gene and Genomes
LDL	Low-density lipoprotein
*Ldlr*	Low-density lipoprotein receptor
LEfSe	Linear Discriminant Analysis for Effect Size
LFD	Low-fat diet
*Lpl*	Lipoprotein lipase
LYST	Lysosomal trafficking regulator
MHC	Major histocompatibility complex
*Mt2*	Metallothionein-2 protein
PBS	Phosphate-buffered saline
PMCA1	Plasma membrane calcium-transporting ATPase
RIN	RNA integrity numbers
RIPA	Radioimmunoprecipitation assay
SCFA	Short-chain fatty acid
SE	Standard error
TIGSS	Texas A&M Institute for Genome Sciences and Society
VLDL	Very low-density lipoprotein
